# Poly[aqua-μ-bromido-(μ_2_-5-methyl­pyrazine-2-carboxyl­ato-κ^4^ *N* ^1^,*O* ^2^:*O* ^2^,*O* ^2′^)lead(II)]

**DOI:** 10.1107/S1600536812033776

**Published:** 2012-08-01

**Authors:** Pan Yang, Yan Liu, Hua Yun Li, Bin Ding

**Affiliations:** a Tianjin Key Laboratory of Structure and Performance of Functional Molecules, Tianjin Normal University, Tianjin 300071, People’s Republic of China

## Abstract

In the title coordination polymer, [PbBr(C_6_H_5_N_2_O_2_)(H_2_O)]_*n*_, the Pb^II^ atom is coordinated by one pyrazine N atom, two bridging Br atoms, a water mol­ecule and three carboxyl­ate O atoms. Bridging by the two anions generates a layer structure parallel to (001); the layers are linked by O—H⋯N and O—H⋯Br hydrogen bonds, forming a three-dimensional network. The lone pair is stereochemically active, resulting in a Ψ-dodeca­hedral coordination environment for Pb^II^.

## Related literature

For background, see: Ding *et al.* (2009 ▶).
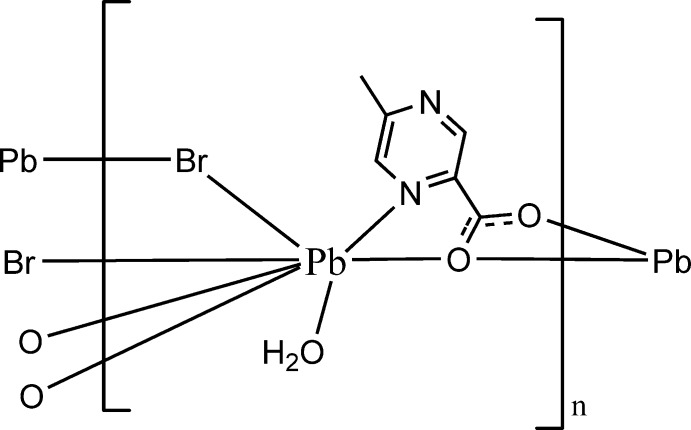


## Experimental

### 

#### Crystal data

[PbBr(C_6_H_5_N_2_O_2_)(H_2_O)]
*M* *_r_* = 442.24
Monoclinic, 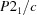

*a* = 7.5493 (10) Å
*b* = 6.6775 (9) Å
*c* = 19.335 (3) Å
β = 92.884 (2)°
*V* = 973.5 (2) Å^3^
*Z* = 4
Mo *K*α radiation
μ = 21.41 mm^−1^
*T* = 296 K
0.15 × 0.14 × 0.13 mm

#### Data collection

Bruker SMART APEX CCD area-detector diffractometer
Absorption correction: multi-scan (*SADABS*; Sheldrick, 1996 ▶) *T*_min_ = 0.142, *T*_max_ = 0.167
5123 measured reflections
1904 independent reflections
1747 reflections with *I* > 2σ(*I*)
*R*_int_ = 0.039

#### Refinement

*R*[*F* ^2^ > 2σ(*F* ^2^)] = 0.033
*wR*(*F* ^2^) = 0.091
*S* = 1.05
1904 reflections
120 parameters
H-atom parameters constrained
Δρ_max_ = 2.26 e Å^−3^
Δρ_min_ = −2.20 e Å^−3^

### 

Data collection: *APEX2* (Bruker, 2007 ▶); cell refinement: *SAINT* (Bruker, 2007 ▶); data reduction: *SAINT*; program(s) used to solve structure: *SHELXS97* (Sheldrick, 2008 ▶); program(s) used to refine structure: *SHELXL97* (Sheldrick, 2008 ▶); molecular graphics: *DIAMOND* (Brandenburg, 1999 ▶); software used to prepare material for publication: *publCIF* (Westrip, 2010) ▶.

## Supplementary Material

Crystal structure: contains datablock(s) global, I. DOI: 10.1107/S1600536812033776/ng5284sup1.cif

Structure factors: contains datablock(s) I. DOI: 10.1107/S1600536812033776/ng5284Isup2.hkl

Additional supplementary materials:  crystallographic information; 3D view; checkCIF report

## Figures and Tables

**Table 1:** Hydrogen-bond geometry (Å, °)

*D*—H⋯*A*	*D*—H	H⋯*A*	*D*⋯*A*	*D*—H⋯*A*
O3—H3*A*⋯N2^i^	0.85	1.97	2.816 (9)	173
O3—H3*B*⋯Br1^ii^	0.85	2.56	3.378 (6)	161

Symmetry codes: (i) 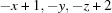; (ii) 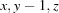.

**Fig. 1.**
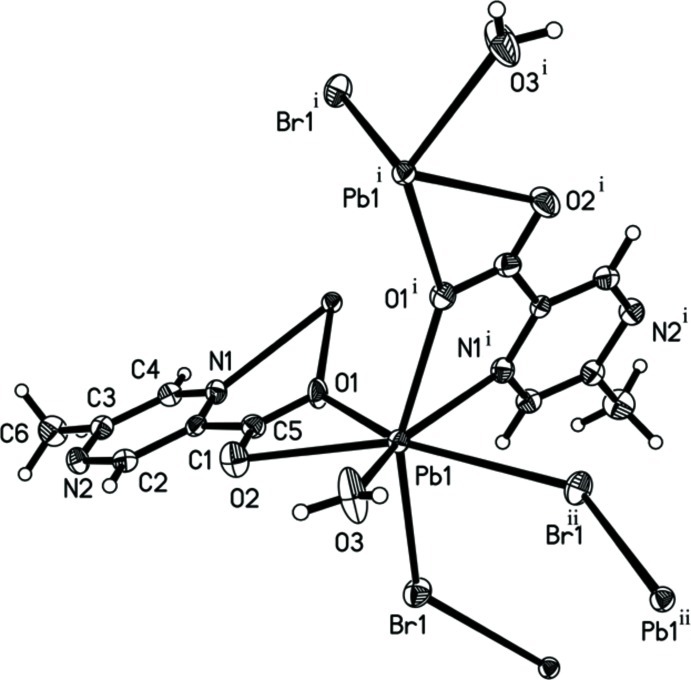
ORTEP plot of a portion of the polymeric structure drawn at 30% probability displacement ellipsoids. Symmetry codes: i: -x + 1, y - 1/2, -z + 3/2; ii: -x, y - 1/2, -z + 3/2.

**Fig. 2.**
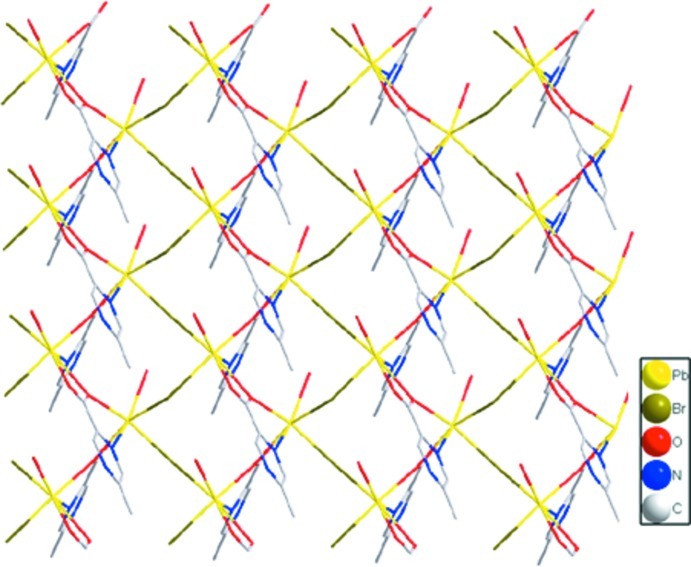
Layer structures.

**Fig. 3.**
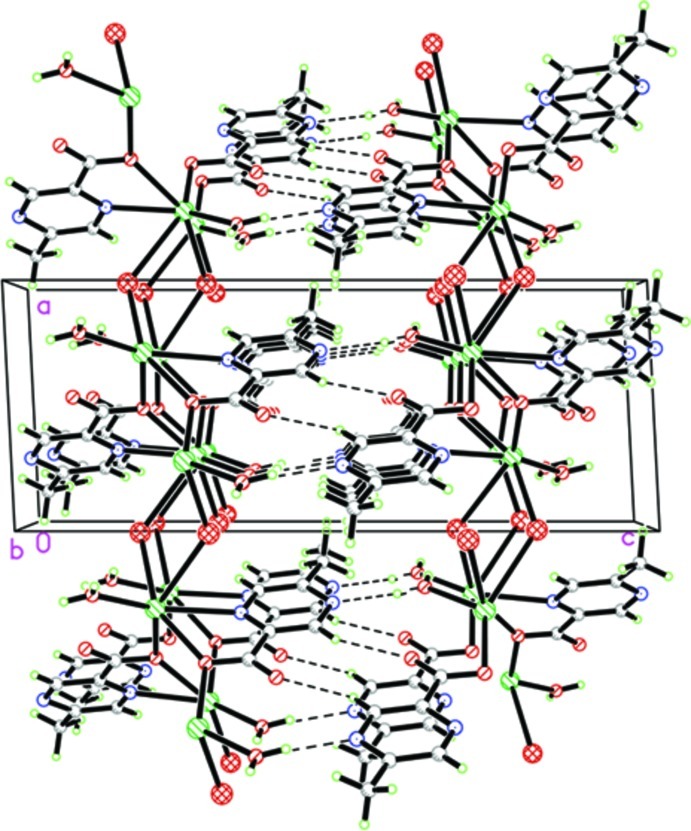
Three-dimensional supramolecular structure. Dashed lines represent O—H···N and weak O—H···Br hydrogen bonds.

**Fig. 4.**
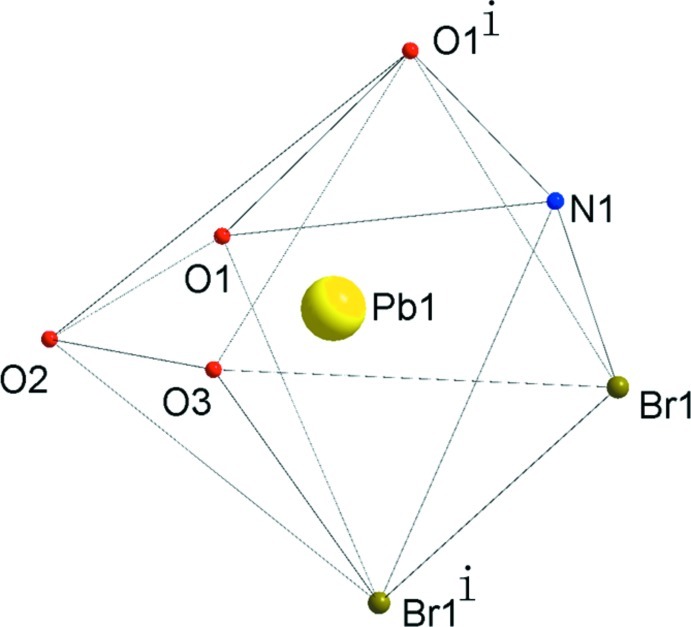
Ψ-Dodecahedral geometry of lead(II).

## supplementary crystallographic information

### Experimental

Lead(II) bromide (73.4 mg, 0.2 mmol), 5-pyrazine-2-carboxylic acid (27.6 mg, 0.2 mmol) and water (15 ml) were sealed in a 25 ml Teflon-lined steel vessel. The mixture was heated heated to 393 K for 5 days . The autoclave was cooled to room temperature at a rate of 10 K h^-1^. Yellow block-shaped crystals were obtained in 60% yield based on Pb.

### Refinement

H atoms were placed in calculated positions as riding atoms attached to non-riding atoms with O—H es of 0.85 Å and C–H 0.93 to 0.96 Å, and with *U*_iso_(H) = 1.2 to 1.5*U*_eq_(O,C). The final difference Fourier map had a peak in the vicinity of Pb and a hole in the vicinty of the same atom.

### Crystal data

**Table:**

[PbBr(C_6_H_5_N_2_O_2_)(H_2_O)]	*F*(000) = 792
*M**_r_* = 442.24	*D*_x_ = 3.018 Mg m^−^^3^
Monoclinic, *P*2_1_/*c*	Mo *K*α radiation, λ = 0.71073 Å
Hall symbol: -P 2ybc	Cell parameters from 3510 reflections
*a* = 7.5493 (10) Å	θ = 3.3–28.2°
*b* = 6.6775 (9) Å	µ = 21.41 mm^−^^1^
*c* = 19.335 (3) Å	*T* = 296 K
β = 92.884 (2)°	Block, colourless
*V* = 973.5 (2) Å^3^	0.15 × 0.14 × 0.13 mm
*Z* = 4	

### Data collection

**Table:**

Bruker SMART APEX CCD area-detector diffractometer	1904 independent reflections
Radiation source: fine-focus sealed tube	1747 reflections with *I* > 2σ(*I*)
Graphite monochromator	*R*_int_ = 0.039
phi and ω scans	θ_max_ = 26.0°, θ_min_ = 3.2°
Absorption correction: multi-scan (*SADABS*; Sheldrick, 1996)	*h* = −9→5
*T*_min_ = 0.142, *T*_max_ = 0.167	*k* = −8→8
5123 measured reflections	*l* = −23→22

### Refinement

**Table:**

Refinement on *F*^2^	0 restraints
Least-squares matrix: full	H-atom parameters constrained
*R*[*F*^2^ > 2σ(*F*^2^)] = 0.033	*w* = 1/[σ^2^(*F*_o_^2^) + (0.0596*P*)^2^] where *P* = (*F*_o_^2^ + 2*F*_c_^2^)/3
*wR*(*F*^2^) = 0.091	(Δ/σ)_max_ = 0.032
*S* = 1.05	Δρ_max_ = 2.26 e Å^−^^3^
1904 reflections	Δρ_min_ = −2.20 e Å^−^^3^
120 parameters	

### Special details

**Table:**

Geometry. All e.s.d.'s (except the e.s.d. in the dihedral angle between two l.s. planes) are estimated using the full covariance matrix. The cell e.s.d.'s are taken into account individually in the estimation of e.s.d.'s in distances, angles and torsion angles; correlations between e.s.d.'s in cell parameters are only used when they are defined by crystal symmetry. An approximate (isotropic) treatment of cell e.s.d.'s is used for estimating e.s.d.'s involving l.s. planes.
Refinement. Refinement of *F*^2^ against ALL reflections. The weighted *R*-factor *wR* and goodness of fit *S* are based on *F*^2^, conventional *R*-factors *R* are based on *F*, with *F* set to zero for negative *F*^2^. The threshold expression of *F*^2^ > σ(*F*^2^) is used only for calculating *R*-factors(gt) *etc*. and is not relevant to the choice of reflections for refinement. *R*-factors based on *F*^2^ are statistically about twice as large as those based on *F*, and *R*- factors based on ALL data will be even larger.

### Fractional atomic coordinates and isotropic or equivalent isotropic displacement parameters (Å^2^)

**Table:**

	*x*	*y*	*z*	*U*_iso_*/*U*_eq_	
Pb1	0.29327 (3)	−0.20899 (3)	0.782606 (12)	0.02328 (17)	
Br1	0.00888 (10)	0.08548 (11)	0.80760 (4)	0.0386 (2)	
O1	0.5113 (7)	0.0867 (8)	0.7933 (3)	0.0351 (12)	
O2	0.4664 (8)	−0.0254 (8)	0.8984 (3)	0.0427 (13)	
O3	0.2211 (10)	−0.5006 (9)	0.8682 (3)	0.067 (2)	
H3A	0.2481	−0.4963	0.9114	0.100*	
H3B	0.1828	−0.6189	0.8608	0.100*	
N1	0.6818 (7)	0.4165 (8)	0.8455 (3)	0.0274 (12)	
N2	0.7181 (8)	0.4728 (9)	0.9872 (3)	0.0322 (13)	
C1	0.6189 (9)	0.2822 (9)	0.8890 (4)	0.0245 (15)	
C2	0.6373 (10)	0.3105 (10)	0.9598 (4)	0.0302 (16)	
H2	0.5925	0.2146	0.9891	0.036*	
C3	0.7783 (9)	0.6113 (9)	0.9431 (4)	0.0298 (15)	
C4	0.7588 (9)	0.5805 (9)	0.8721 (4)	0.0285 (15)	
H4	0.8006	0.6771	0.8423	0.034*	
C5	0.5250 (9)	0.0994 (10)	0.8587 (4)	0.0280 (15)	
C6	0.8627 (14)	0.7958 (11)	0.9749 (6)	0.049 (2)	
H6A	0.7859	0.8517	1.0079	0.073*	
H6B	0.8818	0.8924	0.9392	0.073*	
H6C	0.9743	0.7609	0.9977	0.073*	

### Atomic displacement parameters (Å^2^)

**Table:**

	*U*^11^	*U*^22^	*U*^33^	*U*^12^	*U*^13^	*U*^23^
Pb1	0.0230 (2)	0.0234 (2)	0.0233 (2)	−0.00092 (7)	0.00028 (12)	0.00041 (8)
Br1	0.0362 (4)	0.0338 (4)	0.0451 (5)	0.0095 (3)	−0.0054 (3)	0.0001 (3)
O1	0.036 (3)	0.042 (3)	0.027 (3)	−0.017 (2)	−0.005 (2)	−0.004 (2)
O2	0.056 (4)	0.038 (3)	0.034 (3)	−0.015 (2)	−0.002 (2)	0.009 (2)
O3	0.119 (7)	0.049 (4)	0.031 (3)	−0.039 (4)	−0.019 (3)	0.008 (3)
N1	0.028 (3)	0.026 (3)	0.028 (3)	−0.001 (2)	−0.001 (2)	−0.002 (2)
N2	0.040 (4)	0.030 (3)	0.026 (3)	−0.004 (2)	−0.001 (2)	−0.005 (2)
C1	0.021 (3)	0.026 (4)	0.027 (4)	0.000 (2)	−0.002 (3)	0.000 (3)
C2	0.031 (4)	0.031 (4)	0.029 (4)	−0.001 (3)	0.003 (3)	0.001 (3)
C3	0.030 (4)	0.024 (3)	0.035 (4)	0.000 (3)	−0.003 (3)	−0.003 (3)
C4	0.031 (4)	0.023 (3)	0.031 (4)	−0.002 (3)	0.001 (3)	−0.001 (3)
C5	0.025 (3)	0.030 (3)	0.029 (4)	−0.006 (3)	0.003 (3)	−0.003 (3)
C6	0.059 (6)	0.033 (5)	0.055 (6)	−0.011 (3)	0.004 (5)	−0.008 (4)

### Geometric parameters (Å, º)

**Table:**

Pb1—O1^i^	2.531 (5)	N1—C1	1.334 (8)
Pb1—O1	2.572 (5)	N1—Pb1^iv^	2.631 (5)
Pb1—N1^i^	2.631 (6)	N2—C2	1.340 (9)
Pb1—O3	2.631 (6)	N2—C3	1.353 (9)
Pb1—Br1	2.9688 (8)	C1—C2	1.381 (11)
Pb1—Br1^ii^	3.1190 (9)	C1—C5	1.514 (9)
Br1—Pb1^iii^	3.1190 (9)	C2—H2	0.9300
O1—C5	1.268 (8)	C3—C4	1.388 (10)
O1—Pb1^iv^	2.531 (5)	C3—C6	1.504 (10)
O2—C5	1.230 (8)	C4—H4	0.9300
O3—H3A	0.8501	C6—H6A	0.9600
O3—H3B	0.8510	C6—H6B	0.9600
N1—C4	1.331 (9)	C6—H6C	0.9600
			
O1^i^—Pb1—O1	94.06 (8)	C1—N1—Pb1^iv^	115.1 (4)
O1^i^—Pb1—N1^i^	63.56 (16)	C2—N2—C3	117.6 (6)
O1—Pb1—N1^i^	75.81 (17)	N1—C1—C2	120.8 (6)
O1^i^—Pb1—O3	96.4 (2)	N1—C1—C5	118.2 (6)
O1—Pb1—O3	132.06 (18)	C2—C1—C5	121.0 (6)
N1^i^—Pb1—O3	148.79 (18)	N2—C2—C1	121.6 (6)
O1^i^—Pb1—Br1	153.98 (12)	N2—C2—H2	119.2
O1—Pb1—Br1	86.76 (12)	C1—C2—H2	119.2
N1^i^—Pb1—Br1	91.65 (12)	N2—C3—C4	120.0 (6)
O3—Pb1—Br1	102.33 (18)	N2—C3—C6	116.8 (7)
O1^i^—Pb1—Br1^ii^	82.54 (13)	C4—C3—C6	123.2 (7)
O1—Pb1—Br1^ii^	146.05 (12)	N1—C4—C3	121.8 (6)
N1^i^—Pb1—Br1^ii^	72.46 (13)	N1—C4—H4	119.1
O3—Pb1—Br1^ii^	81.80 (15)	C3—C4—H4	119.1
Br1—Pb1—Br1^ii^	82.410 (17)	O2—C5—O1	124.3 (6)
Pb1—Br1—Pb1^iii^	135.64 (3)	O2—C5—C1	118.7 (6)
C5—O1—Pb1^iv^	121.4 (4)	O1—C5—C1	117.0 (5)
C5—O1—Pb1	98.7 (4)	C3—C6—H6A	109.5
Pb1^iv^—O1—Pb1	139.2 (2)	C3—C6—H6B	109.5
Pb1—O3—H3A	123.1	H6A—C6—H6B	109.5
Pb1—O3—H3B	131.3	C3—C6—H6C	109.5
H3A—O3—H3B	105.1	H6A—C6—H6C	109.5
C4—N1—C1	118.2 (6)	H6B—C6—H6C	109.5
C4—N1—Pb1^iv^	125.1 (4)		
			
O1^i^—Pb1—Br1—Pb1^iii^	13.5 (3)	Pb1^iv^—N1—C1—C5	−15.9 (7)
O1—Pb1—Br1—Pb1^iii^	106.16 (12)	C3—N2—C2—C1	1.4 (10)
N1^i^—Pb1—Br1—Pb1^iii^	30.47 (13)	N1—C1—C2—N2	0.1 (11)
O3—Pb1—Br1—Pb1^iii^	−121.43 (15)	C5—C1—C2—N2	−179.1 (6)
Br1^ii^—Pb1—Br1—Pb1^iii^	−41.59 (4)	C2—N2—C3—C4	−1.4 (10)
O1^i^—Pb1—O1—C5	−126.3 (4)	C2—N2—C3—C6	177.8 (7)
N1^i^—Pb1—O1—C5	172.3 (4)	C1—N1—C4—C3	1.6 (10)
O3—Pb1—O1—C5	−23.9 (5)	Pb1^iv^—N1—C4—C3	−163.3 (5)
Br1—Pb1—O1—C5	79.8 (4)	N2—C3—C4—N1	−0.1 (10)
Br1^ii^—Pb1—O1—C5	151.1 (3)	C6—C3—C4—N1	−179.3 (7)
O1^i^—Pb1—O1—Pb1^iv^	43.5 (3)	Pb1^iv^—O1—C5—O2	−162.2 (6)
N1^i^—Pb1—O1—Pb1^iv^	−17.9 (3)	Pb1—O1—C5—O2	10.0 (8)
O3—Pb1—O1—Pb1^iv^	145.9 (4)	Pb1^iv^—O1—C5—C1	18.3 (8)
Br1—Pb1—O1—Pb1^iv^	−110.4 (3)	Pb1—O1—C5—C1	−169.5 (5)
Br1^ii^—Pb1—O1—Pb1^iv^	−39.1 (5)	N1—C1—C5—O2	−179.9 (6)
C4—N1—C1—C2	−1.5 (9)	C2—C1—C5—O2	−0.7 (10)
Pb1^iv^—N1—C1—C2	164.8 (5)	N1—C1—C5—O1	−0.4 (9)
C4—N1—C1—C5	177.7 (6)	C2—C1—C5—O1	178.8 (6)

Symmetry codes: (i) −*x*+1, *y*−1/2, −*z*+3/2; (ii) −*x*, *y*−1/2, −*z*+3/2; (iii) −*x*, *y*+1/2, −*z*+3/2; (iv) −*x*+1, *y*+1/2, −*z*+3/2.

### Hydrogen-bond geometry (Å, º)

**Table:**

*D*—H···*A*	*D*—H	H···*A*	*D*···*A*	*D*—H···*A*
O3—H3*A*···N2^v^	0.85	1.97	2.816 (9)	173
O3—H3*B*···Br1^vi^	0.85	2.56	3.378 (6)	161

Symmetry codes: (v) −*x*+1, −*y*, −*z*+2; (vi) *x*, *y*−1, *z*.
